# A cost-effective mesocosm framework for reptile research: Design, validation, and practical insights

**DOI:** 10.1371/journal.pone.0337616

**Published:** 2025-11-26

**Authors:** Giulia Simbula, Frederico M. Barroso, Enerit Saçdanaku, Gabriel Ene, Geanina Fănaru, Lekshmi B. Sreelatha, Miruna-Gabriela Vizireanu, Myrto Roumelioti, Nikoletta-Maria Boskovits, Sabina E. Vlad, Prem Aguilar, Miguel A. Carretero

**Affiliations:** 1 CIBIO-InBIO Associate Laboratory, Research Centre in Biodiversity and Genetic Resources, University of Porto, Vairão, Portugal; 2 BIOPOLIS Program in Genomics, Biodiversity and Land Planning, CIBIO, Vairão, Portugal; 3 University of Porto, Vairão, Portugal; 4 Departamento de Biologia, Faculdade de Ciências da Universidade do Porto, Porto, Portugal; 5 Research Center of Flora and Fauna, Faculty of Natural Sciences, University of Tirana, Tirana, Albania; 6 Ovidius University Constanta, Al. Universitatii, Campus B., Constanta, Romania; 7 Association Chelonia Romania, Pascani, Bucharest, Romania; HUN-REN Centre for Ecological Research, HUNGARY

## Abstract

Mesocosms, outdoor replicated ecological experiments within a controlled environment, have become a valuable tool for investigating a broad range of ecological questions across various sub-disciplines. This study presents a medium-sized mesocosm system (MS) designed for individual-level responses to abiotic factors and basic intraspecific interactions in small ground-dwelling reptiles, offering a practical alternative to large-scale facilities for resource-limited settings. Unlike large-scale facilities that are ideal for meta-community dynamics but impractical due to high costs and complexity, this system targets research contexts with limited resources requiring replicated experimental units. To validate the MS design, 16 units were constructed using cost-effective, widely available materials and equipped with a programmable irrigation system. A pilot test using *Podarcis bocagei* lizards provided a preliminary biological evaluation of the design and its suitability in housing wild-caught medium-small reptiles under semi-natural conditions over 7 months. Despite minor maintenance, the MS was resilient to environmental conditions, warranting an expansion to 32 units in subsequent years. Most individuals maintained or recovered body mass during the activity season, and displayed natural behaviours such as basking and foraging. The system’s modularity and adaptability offer a practical reference for ecological studies with similar constraints.

## Introduction

Ecological systems are shaped and influenced by ecological processes [1]. To unravel the underlying mechanisms driving these complex patterns, experimentation is an essential and powerful approach. However, it may face several limitations. Laboratory studies, for instance, allow a high degree of control over various experimental parameters and interactions (e.g., temperature, test duration, dose concentration, etc.) as well as the benefit of increased replicability. On the other hand, their simplified conditions can reduce ecological validity and mundane realism, induce stress due to manipulation and husbandry of the test subject, and often involve small sample sizes constrained by limited space, resources or ethical considerations. In contrast, field studies offer higher ecological realism, but are difficult to replicate and interpret because of the complexity and variability of natural habitats, which involve multiple, often synergistic, causative factors. As a middle ground, mesocosms provide a compelling alternative [2]. Defined as replicated ecological experiments that involve one or multiple species across one or more trophic levels within a controlled environment, mesocosms combine the precise manipulative control of multiple parameters and the replicability of laboratory settings with the environmental realism of field studies, serving as effective validation tools for both approaches [1].

Mesocosms, as semi-natural outdoor enclosures located in situ [2,3], typically attempt to represent a subset of a larger ecosystem. They often involve some form of containment (e.g., a container filled with soil collected from the surrounding environment or water) [4]. Their settings can be easily, although carefully, engineered to suit specific scientific questions, as well as to replicate the structure and function of natural ecosystems, allowing for a high degree of flexibility and creativity in experimental design [1]. Mesocosms have become a valuable tool for investigating a broad range of ecological questions from both aquatic and terrestrial systems across various sub-disciplines, including physiological processes [5], population dynamics [6], community interactions [7], ecotoxicology [8], and behavioural studies [9], among others. Depending on the objective of the research, mesocosm studies can go from simple designs with numerous replicas oriented to recover the individual responses to multiple abiotic factors, to a few, complex structures intended to monitor biotic interactions under limited environmental variation. In any case, trade-offs between complexity and sample size exist due to obvious logistic and financial constraints [10].

In herpetofauna research, a variety of mesocosm designs have been employed to simulate natural habitats and assess ecological processes [11–17]. The majority of these studies tend to focus exclusively on the specific question under investigation, implicitly assuming that the chosen enclosure dimensions are adequate for the species tested, since they are often large-scale infrastructures such as the *Metatron* (https://www.anaee-france.fr/en/infrastructure-services/enclosed-experimentation/terrestrial-metatron/) [18]. However, while this and other larger systems [19–21] provide ideal conditions for studying terrestrial meta-communities and animal movement in response to varying environmental conditions, their complexity, spatial requirements, and high cost make them impractical for smaller-scale applications, resource-limited settings or for analysing interactions between multiple environmental factors. Certainly, in smaller mesocosms, limited space may impose additional constraints on animal behaviour, physiology, and welfare, potentially influencing the outcome of the experiments.

The purpose of this study is to propose a cost-effective medium-sized mesocosm system (MS), designed for individual level studies on medium-small reptiles under semi-natural conditions. Unlike large-scale facilities such as the *Metatron*, our system targets research contexts with limited resources that require replicated experimental units to investigate individual responses to abiotic factors and basic intraspecific interactions (e.g., thermoregulation, reproduction). Specifically, this work provides a detailed, step-by-step construction guide of a MS, accompanied by preliminary validations of the design, using *Podarcis bocagei* (Seoane, 1885) as model species. This species represents an ideal model organism for experimental studies due to several advantages: i) relative low space requirements, ii) short generation times, iii) no parental care; and iv) easy to capture and maintain in captive settings [22].

Changes in body mass and observed physiological behaviours (i.e., basking, borrowing, eggs laying etc) were used as preliminary biological indicators at individual level to provide an initial assessment of the enclosure’s suitability to support the necessary environmental and physiological conditions to host reptiles. Although body condition index is commonly employed to assess health and fitness in reptiles [23], over a period of just a few months, direct changes in body mass may offer a more practical and sensitive measure of the animals’ ability to adapt to the different environments [24].

The ultimate goal of this work is to disseminate the knowledge and insights gained during the development, construction process, maintenance requirements, and biological outcomes of the MS, highlighting its advantages and limitations. By doing so, we aim to offer a practical reference for recreating affordable medium-sized mesocosms for studies focused on individual-level responses in small reptiles facing similar resource constraints.

## Materials and methods

### Mesocosm design

**Fig 1:**
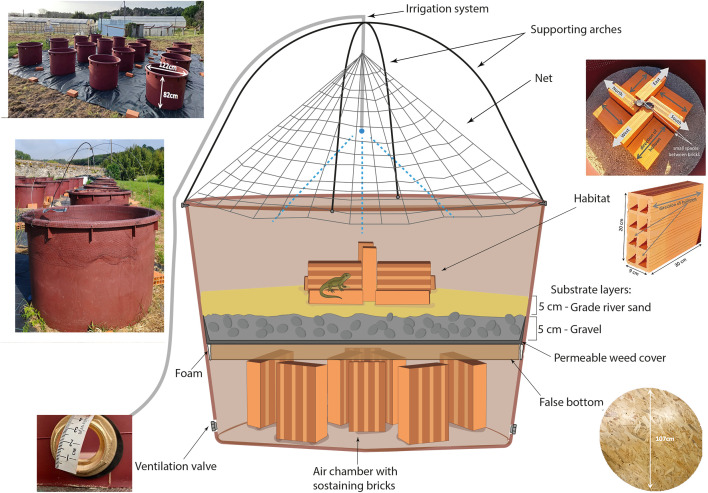
Mesocosm design diagram. Schematic representation of the experimental mesocosm setup ©GS.

The mesocosm structure consisted of a round tub, made out of strong, smooth, solid plastic (Fig 1). Considerations were made regarding the shape of the enclosure: while square shapes would have the advantage of facilitating subdividing the area of the enclosure into a grid system, such a shape would suffer from potential biases related to the presence and inherent directionality of the corners, which may also serve as an escape route. Hence, a round shape was adopted to avoid this. Furthermore, many animals, reptiles included, will exhibit wall-following behaviour [25], changing direction when an obstacle (e.g., a corner or another wall) is found. This could lead to a disproportionate use of space by an animal in a non-round enclosure. An additional consideration that supports both the chosen shape as well as the choice of material (i.e., smooth plastic, as opposed to rough cement) lies in the fact that many reptiles are expert climbers. However round/concave shape with a smooth surface hinders such climbing escape attempts from most reptiles (although not all, e.g., Geckos). Plastic tubs are also usually cheaper and do not heat up as much as their metallic counterparts, and when empty, they can be easily piled and transported without much compromise on structural integrity or durability.

An important advantage of a MS is the capability to deploy several units, allowing for multiple replicates of each treatment. Hence, a MS will usually be comprised of an array of units of mesocosms built to the same standards. Consequentially, several other variables were also taken into consideration before constructing the array of mesocosms used in this study. i) The area needed: a grid-fashion layout was designed to ensure their orderly distribution (250 m^2^ total area occupied by our design). Corridors (~1 m wide) were maintained between rows and columns to allow comfortable access to all parts of each mesocosm and ensure independence of the conditions between units. Additionally, a 2–3 m wide strip around the entire array allowed space to accommodate the irrigation control centre while also serving as a buffer from the surrounding field’s conditions. ii) Probability of external disturbances: each mesocosm was over 200 kg when fully assembled, making it difficult to relocate it (but still possible). Selecting the right site required an assessment of the land’s medium – to long-term use and management plans to ensure that they would not interfere with the MSs’ proper functioning. iii) Since many reptiles thermoregulate through heliothermy, shade/sun exposure is needed, therefore, particular care was devoted to select a location with maximal and homogeneous solar exposure to minimize any potential bias in experiments conducted within the mesocosms due to external facilities (i.e., surrounding walls, buildings, etc.) which could affect thermal availability (e.g., sun/shade exposure, wind exposure). iv) Increasing evidence indicate that hydric requirement is as important as thermal environment for many reptiles, particularly, those species from mesic habitats [26], making the availability of a water source mandatory. The current mesocosm setup relied on an external automated irrigation system, with approximately 50 m of pipes to connect the water source to the irrigation control centre and operated on a powerful (agriculture-grade) water pump. v) Slope of the terrain: the terrain was spread evenly, flattened and compacted to ensure minimal to no slope. This arrangement prevented the mesocosms from shading one another, ensured an even distribution of water flow and pressure across all irrigation sublines, facilitated proper drainage within the system, and avoided issues such as flooding or overly saturated soil conditions. Finally, vi) accessibility: a close proximity between the mesocosms and the laboratory facilitated daily monitoring of the systems as well as a variety of other interventions such as movement of materials with machinery. Once the above factors had been considered, an anti-weed fabric was laid down, stretched across the entire designated area to facilitate access and maintenance around the mesocosms array (Fig 1).

The mesocosm design itself consisted of five different sections: a) an air chamber; b) a false bottom; c) a substrate layer; d) a microhabitat substrate (adaptable to the research goals and target species); and f) the irrigation system (Fig 1). Once placed and stabilised in its final location, two holes were drilled in opposing ends of the tub’s bottom edge to allow for full drainage of water and for adequate ventilation of the wooden false bottom, helping prevent fungal growth. A minimum of eight standard building bricks (30x20x11 cm) were placed vertically and evenly spaced along the bottom of the tub to ensure adequate height of the air chamber, as well as to support and stabilize the false bottom and the entire system. The false bottom was made of OSB plywood board (15 mm thick, 107 cm in diameter), precisely fitted halfway into the tub and drilled with holes for drainage. The board was coated with two layers of wood varnish and topped with a layer of permeable anti-weed fabric to prevent excessive loss of the upper substrates through the drainage holes. Once placed inside the tub, any remaining gaps between the false bottom and the tub walls were filled with non-toxic expanding polyurethane foam. Excess foam was carefully removed, and extra attention was given to avoid dirtying the tub walls with foam, as this could provide a gripping surface for animals to climb out of the mesocosm.

The substrate system used inside the mesocosms consisted of a 2–3 cm gravel layer placed above the anti-weed fabric to ensure drainage and prevent flooding, topped with a medium-grade river sand ~5 cm layer serving as the main substrate. Sand was chosen over soil to limit excessive vegetation growth and enhance drainage. The sand was evenly distributed, forming a gentle slope from the elevated centre toward the perimeter to facilitate water distribution and provide a drier central area. Before adding any microhabitat (see Pilot test below), the mesocosms were left to settle for a few days, subject to daily irrigation treatment. This allowed the substrate to compact and adjust, thus revealing any holes or crevices that would need addressing.

Four small holes were drilled on the top side ledges of the tubs at 90° intervals. Two metal wires were then passed through opposite holes in order to create arches across the tub, secured at the top by zip ties. These arches supported the sprayers of the irrigation system as well as a fine, malleable plastic net (mesh size = 18 mm). The net was essential to prevent the entry of potential predators (e.g., cats, birds, snakes) and was secured using small screws (n = 10–12) partially inserted into the tub ledge sides to act as hooks. This arrangement facilitated the partial elevation of the net when necessary for short maintenance operations or for the release or removal of animals.

**Fig 2:**
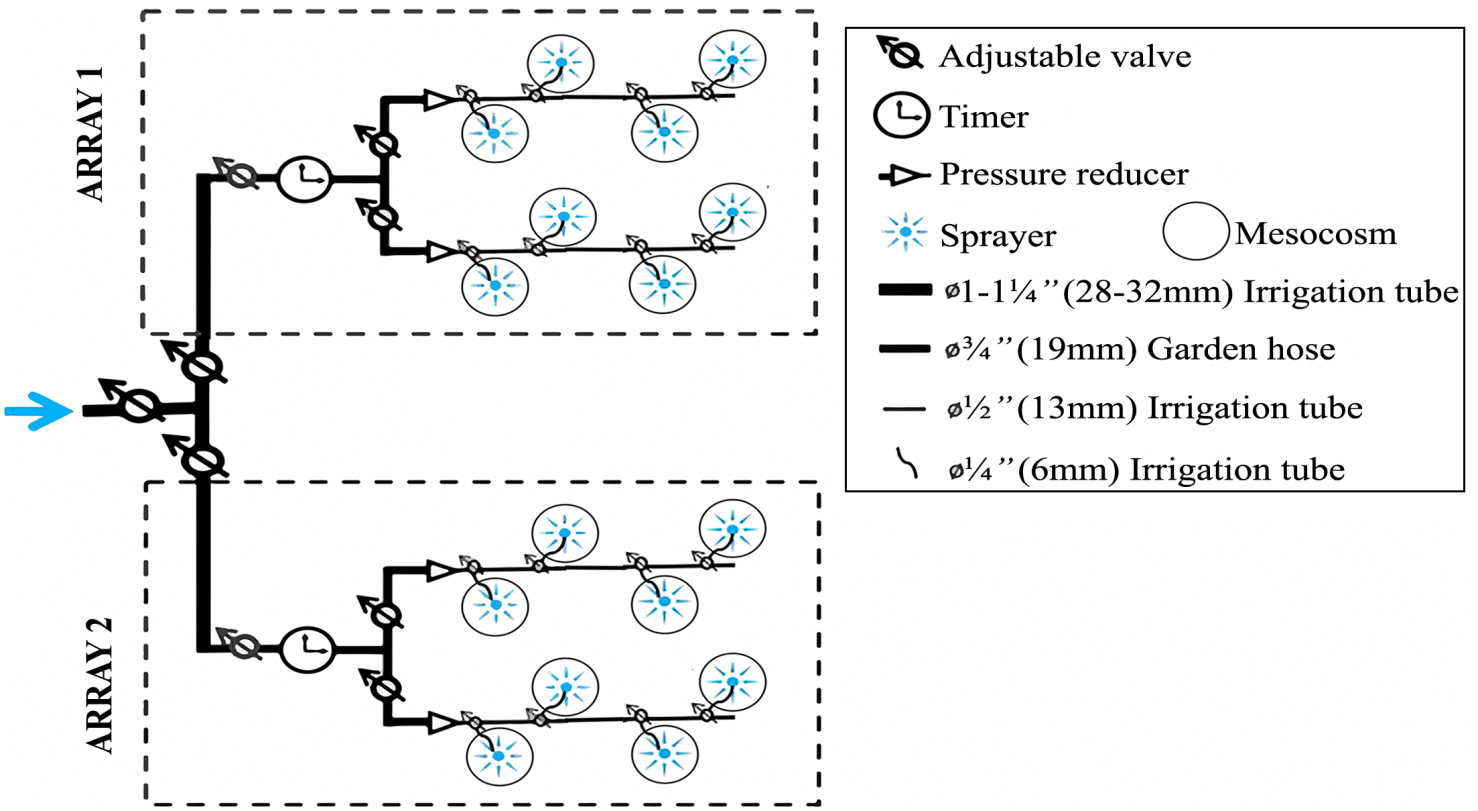
Mesocosm irrigation system. Conceptual diagram of the mesocosms’ irrigation system.

The irrigation system was built to provide four different potential water treatments: the 16 mesocosms were divided into two arrays with two rows of four tubs each (Fig 2). From the main tube, water flowed into two sub-lines within each array, which then branched into smaller irrigation tubes connected to sprayers positioned above each mesocosm. Additionally, each array was equipped with a timer to automate watering, pressure reducers to maintain a consistent and safe water flow, and adjustable valves at every subdivision of the system to ensure even flow between each branch and to facilitate maintenance.

A general inspection of the mesocosm structure was conducted every three days, with repairs and management actions carried out if necessary (i.e., control and eradication of vegetation to keep it below the upper edges of the mesocosm, and removal of incipient ant colonies).

### Pilot test

Sixteen MS were assembled in BIOPOLIS-CIBIO infrastructure (Vairão, Portugal), twelve of which were populated with three adult *P. bocagei* lizards (1 male and 2 females each) locally-caught, housed from May to November 2022, encompassing both reproductive and post-reproductive seasons [27]. This wall lizard species is native to Northwestern Iberia, restricted to mesic environments and quite abundant in the study area [22]. The remaining four MS were left unpopulated to assess the system’s ability to naturally attract insects.

In each mesocosm, eight terracotta construction bricks (30x20x9 cm) with a grid-like (2x4) hollow interior were used to create a suitable habitat, providing structural complexity, refuges, and basking platforms (Fig 1). To ensure uniformity across mesocosms, the bricks were similarly arranged in variate spatial directions, combined with the alternating vertical and horizontal placement, producing a dynamic pattern of rotating shadows throughout the day. This easily replicable arrangement was made to create a predictable thermal heterogeneity in the habitat, allowing the animals to exploit different microclimates as needed. The irrigation system was set to turn on automatically twice a day for 15 minutes (morning 09:00 a.m. and evening 05:00 p.m.).

Lizards were caught from the surrounding area of the mesocosms by noose or hand, sexed using secondary sexual characters [22], measured from snout to vent by a calliper (SVL; precision 0.01 mm), weighted (Digital scale, precision 0.0001 g), photographed by a digital portable scanner (CanoScan LiDE 400), assigned to a specific ID, and randomly distributed in the mesocosms. The reproductive condition of females was evaluated by ventral palpation to detect enlarged ovarian follicles or oviductal eggs. Other reproductive signs (i.e., copulation marks or lateral skin folds) were also noted [27]. Since it quickly became evident that the number of insects naturally entering the mesocosm was insufficient to ensure adequate feeding, lizards were supplemented with two to three crickets (*Achaeta domesticus*) every two days. Daily observations were made to assess the status of the animals, noting their presence, any escapes, or instances of death. Additionally, the mass of each animal was also measured monthly (48 hours after the previous supplemental feeding and just before the next scheduled one), in order to monitor the changes in body mass over time.

This research followed the ethical guidelines of the University of Porto (Portugal). Lizard capture and handling permits were provided by *Instituto da Conservação da Natureza e Florestas* (ICNF, Portugal; permit numbers 560–564/2022/CAPT).

### Statistical analysis

To document the factors driving differences in *P. bocagei* body mass, a generalised linear mixed model (GLMM) was applied using the *lmer4* package in R (version 4.2.2, [28]), considering lizards’ body mass as dependent variable, and sex, experiment duration (months), and their interaction as fixed factors. Since the number of individuals per mesocosm occasionally decreased due to mortality or disappearance (see Results), we also included the number of lizards present at each weighing session as a fixed effect. Mesocosm identity was initially included as a random effect, but its variance was estimated as zero. Therefore, the final models retained only individual as the random effect. Because gravid females might be expected to differ in body mass, we tested pregnancy state (binary gravid/non-gravid) in a preliminary models restricted to females. However, pregnancy effect was inconsistent, driven by its uneven distribution across months (only May–June; S1 Fig.). Therefore, this factor was not retained in the final model (all dataset with male and female together). Post-hoc pairwise comparisons were conducted using the *emmeans* package [29].

## Results

### Mesocosm design

The design and structure of the mesocosms proved to be generally efficient, robust, and resilient to various weather conditions (S2 Fig.). Throughout the entire experimental season, all mesocosms remained fully functional. Only a few minor management interventions were necessary, namely: i) small gaps were observed in some of the mesocosms between the plastic containers and the sand, which were promptly sealed with foam. ii) The irrigation system functioned properly; however, occasional adjustments (e.g., cleaning the nozzle, adjusting the pressure) were required at the sprinkler ends to ensure optimal performance. iii) The protective netting had to be carefully repositioned each time to prevent the lizards from escaping.

### Pilot test

**Fig 3:**
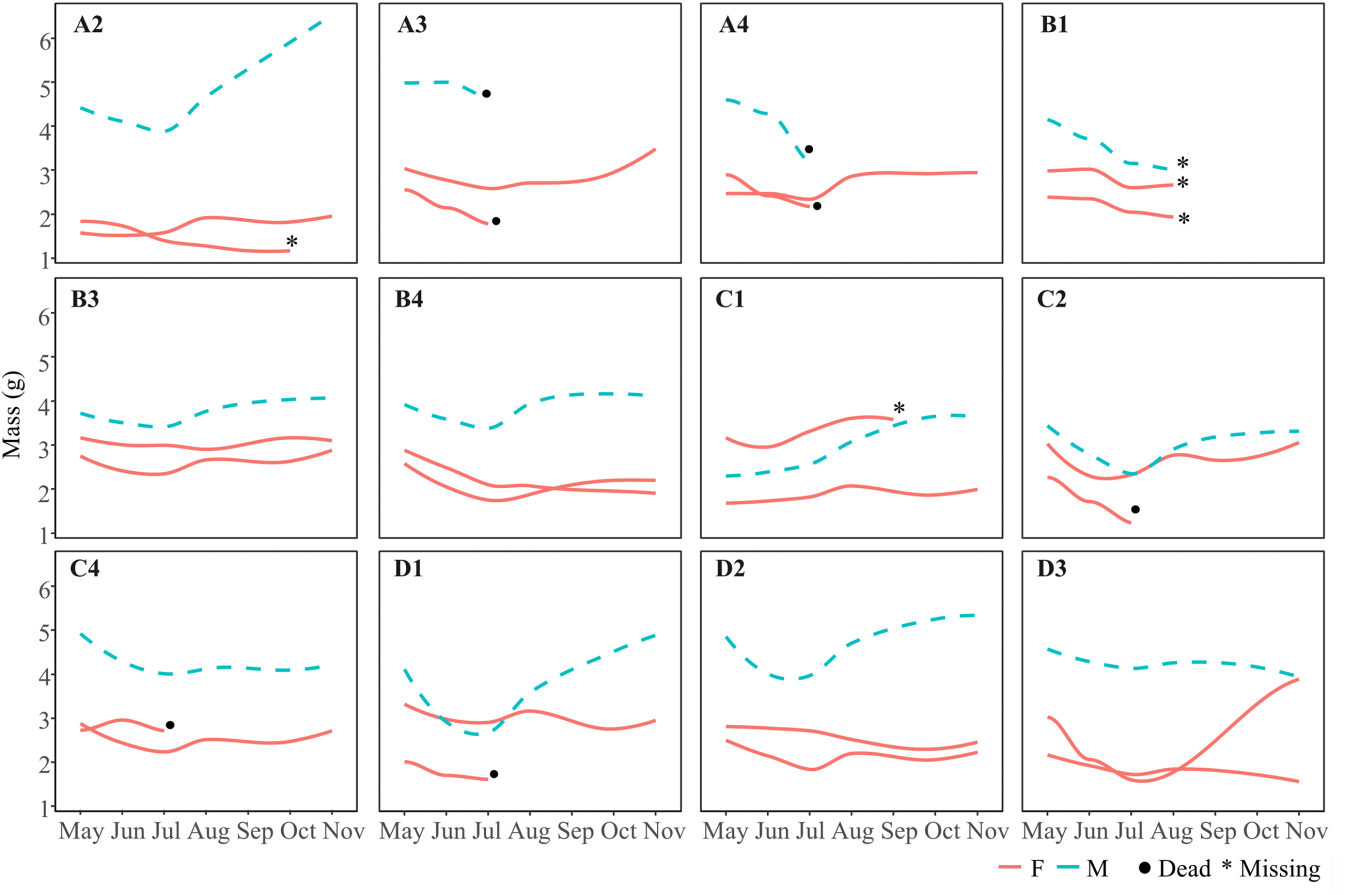
Lizards overall mass change over experimental time in the mesocosms. Mesocosm ID in bold. Red lines and dash blue ones represent respectively females and males’ individuals. Asterisk symbol (*) = missing animals, black dot = dead animals.

Overall, 36 adult lizards (12 males and 24 females) were successfully housed in the mesocosms. Seven animals were found dead (2 males and 5 females) and five went missing (1 males and 4 females) over the course of the seven months trial (Fig 3). Animals were observed engaging in basking, feeding, drinking, and social interactions.

The GLMM analysis revealed a significant effect of most the factors (Table 1). Body mass showed a significant seasonal variation, decreasing in summer and increasing in autumn in both sexes. However, males exhibited a steeper increase than females, surpassing their initial body mass in the later months, whereas females showed a more moderate increase after the initial decline (Fig 3, Table 1).

**Table 1:** (A) Results from the generalised linear mixed model; (B) estimated marginal means (emmean ± SE) per month and sex, and (C) only significant post-hoc Tukey pairwise comparisons for each sex (p values <0.05). n_lizards = number of lizards present in the mesocosms at each weighting occasion; F = females; M = males.

A	Effects	SS	MS	*df*	*F*	*p*
	Sex	8.757	8.757	1	75.486	< 0.001
	Month	14.277	2.379	6	20.513	< 0.001
	n_lizards	0.152	0.152	1	1.309	0.254
	Sex*Month	5.144	0.857	6	7.390	< 0.001
						
B	**Month**	**Females**			**Males**	
	May	2.62 ± 0.12			4.17 ± 0.17	
	June	2.41 ± 0.13			3.88 ± 0.18	
	July	2.19 ± 0.12			3.46 ± 0.17	
	August	2.39 ± 0.13			3.95 ± 0.17	
	September	2.28 ± 0.14			4.22 ± 0.19	
	October	2.35 ± 0.13			4.42 ± 0.17	
	November	2.56 ± 0.14			4.53 ± 0.19	
						
C	**Contrast**	**Sex**	**Estimate**	**SE**	***t***	***p***
	May-July	F	0.432	0.07	5.904	< 0.001
	May-September	F	0.344	0.107	3.225	0.024
	July-November	F	−0.365	0.106	−3.446	0.011
	May-July	M	0.705	0.101	6.951	<0.001
	June-July	M	0.422	0.123	3.435	0.012
	June-October	M	−0.535	0.135	−3.968	0.002
	June-November	M	−0.641	0.155	−4.132	0.001
	July-August	M	−0.483	0.108	−4.625	0.002
	July-September	M	−0.758	0.137	−5.518	< 0.001
	July-October	M	−0.958	0.113	−8.447	< 0.001
	July-November	M	−1.062	0.137	−7.739	< 0.001
	August-October	M	−0.475	0.113	−4.184	< 0.001
	August-November	M	−0.579	0.138	−4.195	< 0.001

## Discussion

The mesocosms designed for this study offer a medium sized, adaptable, and modular solution suitable for addressing both short and medium-term individual-level questions in a semi-natural setting. After one test season, the system proved to be: (i) self-sufficient in terms of water supply, featuring an automated and programmable irrigation system; and (ii) well-suited to housing 2–3 individuals per mesocosm of small, ground-dwelling lizard species (e.g., *Podarcis bocagei*) over extended periods (weeks to months).

Our mesocosm model strikes a balance between being spacious enough to allow natural behaviours in ground-dwelling reptiles (i.e., basking and burrowing) and being cost-effective for studies requiring replication (approximately 215€ per mesocosm). Moreover, the MS is easy to set up using widely available components, most of which can be sourced from agricultural or landscaping suppliers. It may be added that these mesocosms can be even transported with the help of agricultural machinery (e.g., a small tractor). This practicality further highlights its value in contexts where financial or technical resources are limited, or where the research question does not justify investing in advanced or larger facilities.

A key feature of our MS is its modular irrigation system, with independent valves at each stage, allowing for precise pressure control, easy isolation of specific sections, and localized maintenance without the need of shutting down the entire system. This modularity enhances the system’s adaptability, making it suitable for a wide range of experimental designs and semi-natural, medium-term research on reptiles. An additional advantage is the system’s versatility to either host other species of lizards by simply adjusting the hardscape (i.e., substrate type, brick/hide size and layout) or to fit the needs of aquatic species (e.g., amphibians, terrapins) given some fundamental adaptations, for which the system is already pre-prepared to accommodate (e.g., adding a water overflow system to maintain the level, quality and temperature of the water).

Despite these advantages, certain limitations warrant consideration. i) The substantial weight of the tanks (around 200 kg per unit) imposes constraints on the system mobility once fully assembled (although possible given access to adequate machinery). (ii) The layered design presents challenges for maintenance; in the event of malfunctions occurring in the lower strata, resolution may necessitate partial disassembly of the system. Careful attention must be given during the construction process to minimize the risk of complications. iii) The relatively small size of the mesocosm, which based on a partial survey in literature appears to be among the smallest enclosures used in comparable experimental studies, limits the maximum size and number of individuals that can be maintained within each unit. Increasing the number of mesocosms, and thereby replication, could be a straightforward solution for addressing sample size, if emphasis is put on recording individual responses, although this is strongly dependent on available space and budget. Overall, these MS are more suitable for controlled experiments focusing on specific mechanisms rather than for large-scale ecological dynamics. iv) The scarce number of insects naturally entering the mesocosm, making them not self-sufficient in terms of prey availability. Lizards had to be additionally fed with commercially obtained crickets every 48 hours. This procedure inevitably may reduce ecological realism and narrow down the range of questions that can be addressed with this set-up, particularly those related to natural foraging behaviour and diet composition. On the other hand, controlled feeding might ensure standardized prey availability across individuals and replicates, thereby reducing uncontrolled variation in food intake. In the further deployments of the set up, we tried to address this issue by manipulating level of vegetation growth inside the mesocosms as an attractant and habitat for naturally occurring invertebrate prey. However, this came at the cost of increased habitat/structural variability between mesocosm units, as well as possible decreased control over food intake by the test subjects. Depending on the research question, this trade-off between ecological realism and experimental control may represent either a constraint or an advantage of the system.

Regarding the pilot study, we recorded seven animal deaths over the course of the seven months trial. The deceased individuals showed no external abnormalities or signs of illness. While every effort was made to maintain optimal housing conditions and minimize stress, a certain level of mortality is, regrettably, not uncommon in captivity, particularly when dealing with wild-caught or recently acclimated individuals. Factors such as capture stress, environmental changes, and individual physiological variability can all contribute to unexpected mortality [30,31]. Nevertheless, the majority of the lizards tested did not exhibit significant poor body mass or other apparent adverse effects, as indicated by the full recovery of body mass, particularly in the males after summer. This pattern aligns with known physiological traits of the genus *Podarcis*, which exhibits pronounced sexual dimorphism and seasonal variations in body mass, with a sharp decline in mass until June, especially for males, followed by a notable increase peaking in November [27]. These patterns are often coupled with the reproductive cycles in both sexes [27]. For instance, in mature females an inverse relationship between follicular development and fat body volume is well demonstrated in this and many other species of lizards [30,32]. Additionally, the burrowing behaviour of *Podarcis* lizards may help explain the disappearance of some individuals during the study. These lizards are known to dig into the substrate both for thermoregulatory purposes and, in the case of females, to create nests and lay eggs in sandy soil. Despite continuous efforts to promptly detect and repair any potential damage, small gaps or imperfections in the enclosure could have allowed some lizards to access deeper layers of the substrate or even escape from view entirely. Given their cryptic nature and strong substrate interaction, it is plausible that certain individuals were not truly lost but simply went undetected during regular checks. Moreover, this burrowing behaviour initially posed a challenge for egg detection. It required lifting all hardscape elements and thoroughly sifting through the underlying substrate, often involving digging, which was highly disruptive to the habitat and likely stressful for the lizards. To address this and to encourage females to lay their eggs in more accessible and manageable locations, we introduced designated nesting areas, specifically plastic stackable boxes covered by tiles and filled with a moisturized organic soil [33]. While some females consistently chose the nest box to lay their eggs, allowing for easy egg recovery and generally good clutch conditions (clutch sizes range retrieved: 2–4 eggs, mean 3 ± 0.86 SD; [27]), others continued to lay the eggs directly in the sandy substrate of the mesocosms, where retrieval was more complicated. This variability suggests that nest site selection may be influenced by factors such as box size, substrate texture, microclimatic conditions, or simply individual preference. Ultimately, fine-tuning box dimensions and achieving better moisture stability could improve the reliability of this method. Nevertheless, despite these challenges, the mesocosm environment successfully replicated key physiological conditions necessary for natural incubation. This is underscored by the fact that some individuals hatched naturally within the mesocosms. Although our monitoring of eggs success was limited, since it was not even planned in our initial protocol, these preliminary observations suggest that the system can provide sufficiently stable temperature, humidity, and substrate conditions to promote egg laying and embryonic development. More systematic and carefully controlled studies will be needed to assess reproductive success in detail.

Following this initial study, over the subsequent three years, the setup was expanded by adding 16 more units, for a total of 32 mesocosms. This allowed for larger-scale studies and greater experimental replication. Although the system was left inactive during the winter months, only four of the 32 mesocosms exhibited structural wear requiring different interventions, thus highlighting a reasonable level of durability for an outdoor, low-cost setup. On the other hand, close monitoring, regular maintenance and possible improvements/updates, depending on the specific scientific purpose, remain essential to ensure the efficiency of the system as well as its lifespan. Furthermore, a particularly important aspect for future applications of this MS involves improving biosecurity and containment, especially in view of the occasional disappearance of individuals observed during our trials. While these cases were likely due to the strong burrowing behaviour of *Podarcis* lizards [22], they nonetheless exposed vulnerabilities in the current setup that must be addressed, especially if the system is to be adapted for use with non-native, potentially invasive species. In this context, several containment measures should be considered or reinforced, such as: i) installing deeper and continuous buried barriers along the internal perimeter to prevent digging-based escapes; ii) adding vertical extensions or overhanging structures to reduce the risk of animals climbing out; and iii) incorporating optional netting or roofing systems to fully enclose the top of the mesocosms when necessary. Implementing these measures would significantly enhance the biosecurity performance of the system and make it suitable for a broader range of experimental conditions, including those involving species that require strict containment under environmental or regulatory guidelines.

In conclusion, the system presented fills a practical niche for replicated individual-level studies on small reptiles. The limitations we have identified, particularly regarding containment, food supplementation, and size constraints, offer guidance on scenarios where this system is appropriate and where alternative approaches may be better suited. By transparently sharing both successes and challenges encountered during development, we aim to facilitate informed decision-making for subsequent studies considering similar experimental infrastructures.

## Supporting information

S1 Fig Body mass of female lizards by month and pregnancy status. Points represent individual body mass measurements, with gravid females in black and non-gravid in white. Symbols and error bars indicate mean ± SE for each group. Pregnant females occurred only in May–June, which explains the apparently negative effect of pregnancy observed in the model including month as a covariate (β = −0.20 ± 0.08, F_1,178_ = 5.78, p = 0.017). Without month, pregnancy was not significant (β = 0.02 ± 0.08, F_1,185_ = 0.05, p = 0.83).
(TIF)

S2 Fig Climate overview. Monthly average temperature (°C) and total precipitation (mm) recorded in 2022 in Portugal (weather station n. 85450). Temperature trends are shown as colored lines (mean, max, min), and precipitation as scaled blue bars (right y-axis).
(TIF)
